# Pain in Endometriosis

**DOI:** 10.3389/fncel.2020.590823

**Published:** 2020-10-06

**Authors:** Jessica Maddern, Luke Grundy, Joel Castro, Stuart M. Brierley

**Affiliations:** ^1^Visceral Pain Research Group, College of Medicine and Public Health, Flinders Health and Medical Research Institute, Flinders University, Adelaide, SA, Australia; ^2^Hopwood Centre for Neurobiology, Lifelong Health Theme, South Australian Health and Medical Research Institute, Adelaide, SA, Australia; ^3^Discipline of Medicine, University of Adelaide, North Terrace Campus, Adelaide, SA, Australia

**Keywords:** chronic pelvic pain, sensory afferents, uterus, vagina, inflammation, neuroangiogenesis, peripheral sensitization, hyperalgesia

## Abstract

Endometriosis is a chronic and debilitating condition affecting ∼10% of women. Endometriosis is characterized by infertility and chronic pelvic pain, yet treatment options remain limited. In many respects this is related to an underlying lack of knowledge of the etiology and mechanisms contributing to endometriosis-induced pain. Whilst many studies focus on retrograde menstruation, and the formation and development of lesions in the pathogenesis of endometriosis, the mechanisms underlying the associated pain remain poorly described. Here we review the recent clinical and experimental evidence of the mechanisms contributing to chronic pain in endometriosis. This includes the roles of inflammation, neurogenic inflammation, neuroangiogenesis, peripheral sensitization and central sensitization. As endometriosis patients are also known to have co-morbidities such as irritable bowel syndrome and overactive bladder syndrome, we highlight how common nerve pathways innervating the colon, bladder and female reproductive tract can contribute to co-morbidity via cross-organ sensitization.

## Introduction

Endometriosis is a chronic and debilitating condition characterized by chronic pelvic pain (CPP) and infertility. Endometriosis has a large clinical burden, affecting approximately 1 in 10 women globally (Simoens et al., 2007; Adamson et al., 2010; Nnoaham et al., 2011). As such, endometriosis is associated with significant societal and economic burden that costs the US economy $22 billion annually in lost productivity and direct healthcare costs (Simoens et al., 2007; Adamson et al., 2010; Nnoaham et al., 2011). Despite this burden, the etiology and pathogenesis of endometriosis remains poorly defined, whilst efficacious therapeutic interventions remain limited.

Endometriosis is diagnosed surgically by the presence of endometrial lesions outside of the uterus. However, surgical intervention and diagnosis are usually preceded by bouts of chronic abdominal pain and/or CPP, leading patients to present to their consulting physician. More than 60% of women diagnosed with endometriosis report CPP, with endometriosis patients 13 times more likely to experience abdominal pain than healthy subjects (Ballard et al., 2008). Despite this disease homogeneity, the mechanisms by which endometriosis induces a chronic pain state remain poorly understood (Ballard et al., 2008). Surgical removal of endometrial lesions can be successful in alleviating pain in endometriosis patients, supporting a role of these lesions in chronic pain. However, disparity exists in the correlation between pain severity and lesion score (Porpora et al., 1999). Moreover, chronic pain frequently returns in patients within 12 months of lesion removal, even in the absence of lesion regeneration (Abbott et al., 2004; Shakiba et al., 2008). As such, there is a clear disconnect between the traditional theory that endometriosis associated pain is solely dependent on lesions, and the reality experienced by endometriosis patients throughout the world. This disconnect is further highlighted by the observation that endometriosis patients frequently suffer from a number of clinical comorbidities. Endometriosis is commonly co-diagnosed with bladder and colon disorders that are characterized by sensory dysfunction, such as overactive bladder syndrome (OAB), and irritable bowel syndrome (IBS) (Surrey et al., 2018). These comorbidities are suggestive of a more complex pathophysiology for endometriosis induced pain that cannot be explained by endometrial lesions alone. Growing evidence now indicates that a chronic remodeling of the nervous system occurs in shared sensory neural pathways to induce a state of protracted peripheral and central sensitization and chronic pain in endometriosis patients.

In this review, we summarize the most recent clinical and preclinical research to highlight advances in determining the mechanisms underlying the development of endometriosis associated CPP and chronic abdominal pain. In particular, we highlight the mechanisms occurring in the periphery.

### Pathogenesis

**FIGURE 1:**
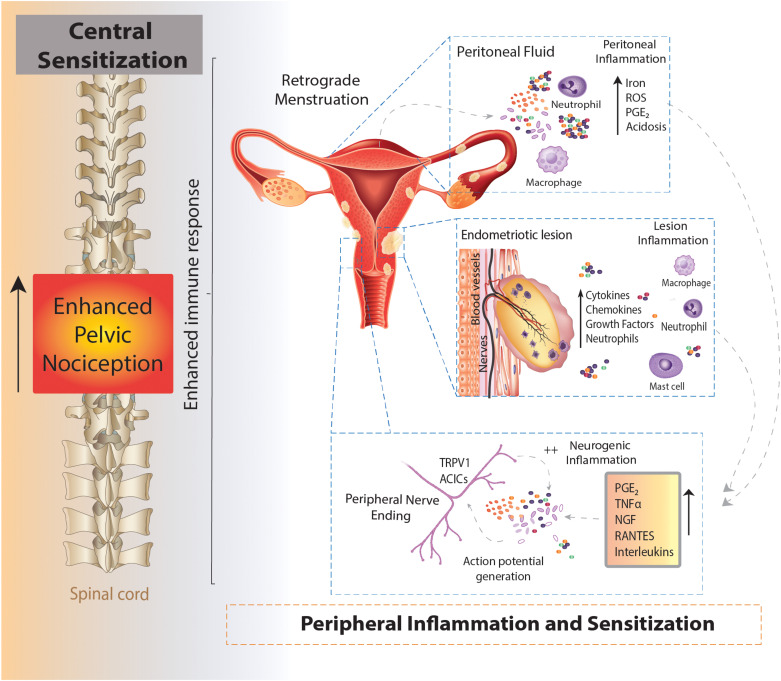
Schematic overview of an enhanced immune response leading to chronic pelvic pain in endometriosis. Endometrial fragments in the peritoneum, originating from the uterus during retrograde menstruation, lead to the production and build-up of iron, reactive oxygen species (ROS), prostaglandins (PGE_2_) and acidosis in the peritoneal fluid (peritoneal inflammation). This immune response is also seen at lesions sites throughout the peritoneal cavity, where the increased production of cytokines, chemokines, growth factors and neutrophils also contribute to an enhanced inflammatory environment present in the peritoneal cavity of women with endometriosis (lesion inflammation). Of these inflammatory mediators, PGE_2_, tumor necrosis factor α (TNFα), nerve growth factor (NGF), RANTES and interleukins (IL) IL-8 and IL-1β are able to directly activate sensory nerve endings and activate a positive feedback loop, further increasing proinflammatory modulator production (neurogenic inflammation). The enhanced stimulation and activation of peripheral nerve endings in the peritoneal cavity (peripheral sensitization) increases the painful stimuli transmitted to the spinal cord, initiating and maintaining chronic pelvic pain (central sensitization).

As an estrogen dependent disorder, endometriosis predominantly effects women of reproductive years aged 15–50 (Giudice and Kao, 2004). Endometriosis is a chronic inflammatory condition, characterized by the migration of uterine endometrial cells into the pelvic cavity, where they form lesions on multiple sites across multiple organs. Retrograde menstruation is the generally accepted mechanism underlying the pathogenesis of endometriosis (Sampson, 1927; Giudice and Kao, 2004). This mechanism was originally proposed in 1927, whereby endometrial fragments migrate from the fallopian tubes into the peritoneal cavity during menstruation (Figure 1). However, retrograde menstruation naturally occurs in 90% of women, but only 10% of women develop endometriosis (Halme et al., 1984b). More recent data show the prevalence of retrograde menstruation is no different in women with or without endometriosis, suggesting a more complex etiology (Kruitwagen et al., 1991; O et al., 2017). Patients with endometriosis typically have more frequent and higher volumes of menstrual flow, shorter menstrual cycle intervals and increased endometrial fragments in the peritoneal cavity (Liu and Hitchcock, 1986; Tal et al., 2019). Once the endometrial debris becomes ectopic, adhesion needs to occur in order to initiate the development of lesions and the induction of endometriosis. While the mechanisms underlying this process remain unclear, it is considered that immune dysfunction and the subsequent inability to effectively clear these fragments enables endometrial lesions to form in the peritoneal cavity (Giudice and Kao, 2004).

Normal menstruation requires the initiation of an innate immune response to provide cyclical breakdown and clearing of endometrial cells from the uterus in a highly regulated process (Critchley et al., 2001). Similarly, in women who don’t develop endometriosis, ectopic endometrial debris is cleared from the peritoneum by an innate immune response characterized by increased local neutrophils, macrophages, natural killer (NK) cells and dendritic cells (O et al., 2017; Symons et al., 2018). Dysregulation in this innate immune response in response to ectopic endometrial tissue has been implicated in endometriosis pathophysiology. Numerous studies have shown increased peritoneal macrophages in response to the infiltration of circulating monocytes in the peritoneal cavity (Halme et al., 1984a; Amaral et al., 2006; Banu et al., 2008), which facilitate the growth of endometrium-like tissue (Oosterlynck et al., 1992; Dmowski et al., 1998; Chuang et al., 2010). In women without endometriosis, tight regulation and control of immune cells clears the way for the next menstrual cycle. However, in women with endometriosis the chronic inflammatory milieu has been linked to the generation of pain (Laux-Biehlmann et al., 2015).

Although retrograde menstruation is the oldest and most widely accepted mechanism, other theories have been proposed in the pathogenesis of endometriosis. Amongst these, metaplasia of specialized cells in the peritoneal tissue into endometrial like cells (Coelomic metaplasia), hormonal imbalance, progesterone resistance, immune dysfunction and inflammation, are all able to facilitate lesion growth and a genetic component that provides bias toward first degree relatives and twins (Sourial et al., 2014). Understanding the pathogenesis of endometriosis and how each of these mechanisms may intertwine is an important area for research. Elucidating risk factors and mechanisms will enable a more widespread understanding of the development of endometriosis and ultimately treatment and prevention of ectopic lesion formation and growth.

### Clinical Diagnosis

The most common symptoms of endometriosis include dysmenorrhea, back pain and chronic lower abdominal pain or CPP that is associated with bladder filling and defecation. As these symptoms mimic those of other diseases or conditions, the clinical diagnosis of endometriosis is complex (Hickey et al., 2014). Despite multiple studies aimed at identifying biomarkers in the blood or urine to facilitate non-invasive biological testing and clinical diagnosis of endometriosis, no biomarkers have been identified as of yet (Nisenblat et al., 2016; Chapron et al., 2019). Clinical examination via palpation of the abdominal and pelvic area, in conjunction with patient history are early stages of the endometriosis diagnosis process, but are only stepping stones to further investigation (Riazi et al., 2015). Non-surgical clinical examination cannot definitively diagnose endometriosis with ultrasound, the most common non-invasive investigation, correctly identifying only ∼10% of cases (Pugsley and Ballard, 2007). Recent advances in medical imaging suggests potential for transvaginal ultrasound and MRI as diagnostic tools for some types of endometriosis, however, the identification of superficial peritoneal lesions is not yet possible as the size of the lesions is below the threshold for detection (Guerriero et al., 2018; Van den Bosch and Van Schoubroeck, 2018; Chapron et al., 2019). Accordingly, endometriosis can only be diagnosed and classified via surgical laparoscopy or laparotomy to confirm the presence of endometriotic lesions (Hickey et al., 2014). Due to the surgically invasive nature of this diagnostic testing, as well as the limited understanding of endometriosis pathology, progression, and risk factors, there is often frequent misdiagnosis and a consequential delay in determining the correct diagnosis. Multiple studies investigating the time between the onset of pain and the diagnosis of endometriosis report a significant delay, with an average of 8 years in the United Kingdom and up to 12 years in many countries including the United States (Hadfield et al., 1996; Arruda et al., 2003; Husby et al., 2003; Nnoaham et al., 2011). Such delays in diagnosis are likely to aid in the progressive chronicity of the disease.

Once surgically confirmed, the stage and extent of endometriosis is categorized via a standardized worldwide classification system into one of four grades, determined by the severity of lesions/adhesions (Taf, 1979; Canis et al., 1997). Although this is the standard and accepted method for classification, there is only a weak correlation between the graded severity of morphological characteristics and the intensity and character of pain symptoms (Vercellini et al., 2007; Hsu et al., 2011). As diagnosis and classification of endometriosis evolves, it has become clear that it is a chronic and systemic disease with inadequate understanding, and diagnosed stage is not indicative of patient suffering (Adamson, 2011; Johnson et al., 2017). The location of ectopic lesions can vary dramatically between patients and are found predominantly on the peritoneum, but also adhered to organs, including ovaries, vagina, fallopian tubes, bladder, colon and small intestine (Chapron et al., 2003; Stegmann et al., 2008). Whilst pain is often experienced from the reproductive organs, pain can also originate from those organs in close proximity, such as the bowel and bladder. Accordingly, patients with endometriosis exhibit significant comorbidity with chronic abdominal pain and CPP disorders such as interstitial cystitis/painful bladder syndrome (70–80% of endometriosis patients) (Chung et al., 2005; Tirlapur et al., 2013) and IBS (50–70% of endometriosis patients) (Jess et al., 2012; Schomacker et al., 2018). Although lesions can be removed or ablated as an initial treatment for endometriosis associated CPP, additional treatments or further surgery are often required. Whilst surgery, hormonal and non-hormonal pharmaceutical agents (discussed in greater detail below) are used to try and resolve CPP (Table 1), complete eradication of endometriosis-associated pain is rarely possible.

**TABLE 1:** Current approaches for CPP in endometriosis.

*Class*	Type	Mechanism	Side effects
*Hormonal*	GnRH agonists Aromatase inhibitors Contraceptive Pill	Modulation of hormone signaling and prevention of menstruation	Hypoestrogenic – decreased bone density, mood changes and breast atrophy Not viable with pregnancy
*Non-hormonal*	NSAIDs Opioids	Targets inflammation	Often ineffective Exhibit toxicity Tolerance, dependence, constipation and addiction
*Surgical*	Lesion excision or ablation Partial or complete Hysterectomy	Target lesions or uterus as site of endometriosis	Invasive surgery with significant complication rates Early menopause Not always viable with pregnancy (hysterectomy)

## Mechanisms Underlying Endometriosis Induced Pain

The majority of research into the mechanisms underlying pain in endometriosis has focused on the endometrial lesions and adhesions as the primary source of endometriosis-associated pain. Although lesion specific pain is apparent, and undoubtably essential for the induction of endometriosis induced pain, lesion removal does not provide pain relief in all cases, with reports suggesting 20–28% of patients do not have pain relief following surgery (Sutton et al., 1994; Abbott et al., 2004). Furthermore, only a marginal association exists between endometriosis stage (defined by lesion morphology) and the severity of pelvic symptoms (Vercellini et al., 2007), suggesting additional complex mechanisms are involved. More recent understanding of the mechanisms underlying the development of a chronic pain state in endometriosis implicates cyclical bleeding from lesions and subsequent inflammation at both lesion sites and in the peritoneal cavity. These proinflammatory responses then result in sensory nerve activation and altered activation of nociceptive pathways (Table 2) (Morotti et al., 2017; Patel et al., 2018; Zheng et al., 2019).

**TABLE 2:** Pain mechanisms in endometriosis.

	Site	Mechanism	Key players in pain
*Inflammation*	Peritoneal fluid and endometrial lesions	Retrograde menstruation and cyclical bleeding at lesion sites	Activation of an innate immune response increases inflammatory and nociceptive cytokines/chemokines (TNFα interleukins IL-8 and IL-1β) (Barcz et al., 2000), ROS, growth factors (NGF and VEGF) (Donnez et al., 1998), neutrophils and prostaglandins (PGE_2_) (Sacco et al., 2012; Králíèková and Vetvicka, 2015)
*Neurogenic inflammation*	Sensory nerves	Build-up of pro-nociceptive environment can act directly on sensory nerve fibers	Degraded tissue by-products including ROS, PGE_2_ and acidification can activate sensory nerves (Reeh and Steen, 1996; Holzer, 2011). Positive feedback loop maintains inflammation by releasing further proinflammatory modulators including SP and CGRP (Tokushige et al., 2006b).
*Peripheral Sensitization*	Peripheral sensory nerves	Neuroplasticity of peripheral sensory nerves	Persistent inflammation can cause structural and synaptic changes occur to shift the neuronal function into a more sensitized state (Brierley and Linden, 2014). The abundance of inflammatory molecules in the peritoneal fluid, including glycodelin, ROS, TNFα, NGF, and PGE_2_ may contribute to this (Chiu et al., 2012).
*Central Sensitization*	Central nervous system (CNS)	Long term changes in CNS signaling	Persistent nociceptive barrage leads to a long-lasting central sensitization of sensory afferents, evoking long term changes in pain processing or ‘memory’ (Bajaj et al., 2003; Woolf, 2011).
*Cross-organ Sensitization*	Sensitized afferents across multiple organs	Sensitized afferents from one organ induce sensitization of the afferents innervating another organ	Visceral afferents converge into similar areas of the spinal cord providing opportunity for the sensitization of neighboring cells due to spatial location (Ge et al., 2019)

### Inflammation

In the uterus, the natural breakdown and elimination of endometrial tissue during menstruation is a highly regulated response to falling levels of estrogen and progesterone, acting to remove menstrual debris from the uterus, to make way for another cycle of endometrial regeneration (Berbic and Fraser, 2013). Carried out by the innate immune system, this process recruits a large number of immune cells, including neutrophils, macrophages and natural killer cells (NKs) to facilitate menstrual breakdown. The endometrial lining undergoes apoptosis and necrosis and is then finally shed (Berbic and Fraser, 2013). The programmed cell death during menstruation releases numerous cellular products, including iron, reactive oxygen species (ROS), prostaglandins and a family of damage-associated molecular patterns (DAMPs) amongst others (Laux-Biehlmann et al., 2015). When retrograde menstruation occurs endometrial fragments adhere and form lesions within the peritoneum, where they remain endometrial in nature, expressing estrogen receptors and undergoing cyclical inflammatory and menstrual events (Brosens, 1997; Burney and Lathi, 2009). Cyclical bleeding in response to sex hormones, which naturally occurs in the uterus during menstruation, can also be seen within the peritoneal cavity at the site of endometrial lesions (Halme et al., 1984a). In endometriosis, menstrual by-products also arise from extra-uterine lesions and are released into the peritoneal cavity where they initiate immune responses (Table 2 and Figure 1) (Laux-Biehlmann et al., 2015). Not only does the attempted breakdown of existing endometriotic lesions and residual retrograde menstruation in the peritoneal cavity activate an immune response, but the amplified inflammatory milieu leads to a build-up of iron, ROS, prostaglandin E2 (PGE_2_) and acidosis (Reeh and Steen, 1996). This immune response is evident at lesion sites, with increased inflammatory cytokines/chemokines, growth factors, neutrophils and prostaglandins found within the peritoneal cavity of endometriosis patients (Donnez et al., 1998; Barcz et al., 2000; Sacco et al., 2012; Králíèková and Vetvicka, 2015). Of these mediators, PGE_2_, tumor necrosis factor-α (TNFα), nerve growth factor (NGF), Regulated on Activation Normal T cell Expressed and Secreted (RANTES, also known as C-C chemokine ligand 5: CCL5), and interleukin (IL) IL-8 and IL-1β are all elevated within the peritoneal fluid of endometriosis patients who reported CPP pain (Ryan et al., 1995; Barcz et al., 2000; Bedaiwy et al., 2002; Scholl et al., 2009). Crucially these mediators are all able to directly activate sensory nerve endings, suggesting inflammatory mechanisms may be important in endometriosis associated (Wu et al., 2017; Liang et al., 2018). A recent study showed that cytokine analysis of peritoneal fluids could differentiate women diagnosed and stratified laparoscopically with ovarian endometrioma, peritoneal endometriosis, or deep infiltrating endometriosis. This suggests that certain cytokine signatures could be driving different biological signaling events and immune responses in these patients (Zhou et al., 2020).

In addition, ROS induce oxidative modification of proteins and significantly higher levels of protein oxidative stress markers are found within the peritoneal fluid of women with endometriosis (Santulli et al., 2015). The targeting of oxidized proteins with oral antioxidants, including vitamin E and vitamin C, significantly reduces reported chronic pain in women with endometriosis compared to placebo following 8 weeks of treatment (Santanam et al., 2013). This treatment approach also decreased inflammatory markers within the peritoneal fluid (Santanam et al., 2013). Oxidized proteins, and the subsequent activation of nociceptors, has been suggested to promote endometriosis associated pain although the underlying mechanism remains unclear (Ray et al., 2015; Wright et al., 2017).

Overall, the resulting increase in extra-uterine debris, heightened by further retrograde menstruation, induces an enhanced inflammatory response within the peritoneum and presents an opportunity for the interaction of these immune cells with sensory nerves to induce CPP (Asante and Taylor, 2011). Long term exposure to proinflammatory cytokines is proposed to activate and sensitize sensory nerves present in endometriotic lesions, initiating the transfer of pain to the central nervous system (CNS) (Laux-Biehlmann et al., 2015). The activation of sensory nerve fibers to generate and convey pain to the CNS is a vital step in the pain processing pathway, and contributes to other forms of chronic visceral pain (Berkley, 2005; Brierley and Linden, 2014; de Groat et al., 2015; Grundy and Brierley, 2018). Whilst this cyclical inflammation can exacerbate pain during menstruation, non-cyclical pain is also experienced by many women with endometriosis (Vercellini et al., 2007). This suggests further mechanisms, independent of cyclical events, contribute to CPP in endometriosis.

### Neuroangiogenesis

**FIGURE 2:**
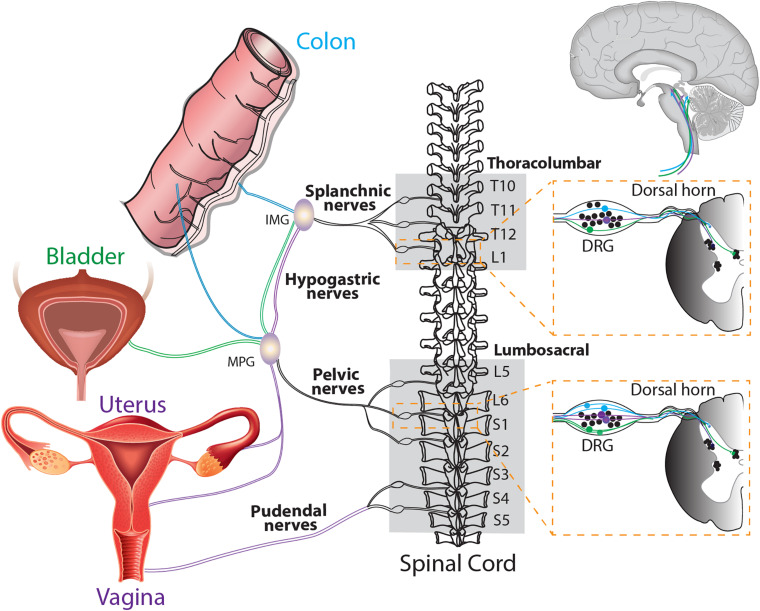
Schematic overview of the sensory innervation of the colon, bladder and uterus/vagina allowing for cross organ sensitization. The colon is innervated by spinal afferents within the splanchnic and pelvic pathways. These spinal afferents can travel via the splanchnic or pelvic nerves (with some afferents traversing the hypogastric nerve) and have cell bodies located within the thoracolumbar and lumbosacral dorsal root ganglia (DRG). The bladder is also innervated by both splanchnic and pelvic pathways (de Groat et al., 2015). The central terminals of colon and bladder afferents terminate within the dorsal horn of the thoracolumbar and lumbosacral dorsal horn (Harrington et al., 2012, 2019; Grundy et al., 2018). Afferents innervating the uterus and vagina also share the splanchnic and pelvic pathways, whilst the vagina is also innervated by the pudendal nerves, with cell bodies within the lumbosacral DRG and central terminals within the lumbosacral dorsal horn (Berkley, 2005). The central terminals of colon, bladder, uterus and vaginal afferents synapse onto second order dorsal horn neurons in the spinal cord, which ultimately transmit signals to the brain. Accordingly, these different visceral organs share common nerve pathways to the DRG and spinal cord, allowing the potential for cross-organ sensitization. TL, Thoracolumbar; LS, Lumbosacral; IMG, Inferior Mesenteric Ganglion; MPG, Major Pelvic Ganglion.

Pain relies on the existence of sensory transduction pathways linking peripheral stimuli to the spinal cord for processing and the brain for perception (Figure 2). However, endometrial fragments do not have an intrinsic sensory nerve supply connected to these pathways. For endometrial lesions to induce chronic pain, the development of new sensory nerves to convey these signals needs to occur. A number of studies have now shown that once endometrial fragments adhere to a peritoneal location and become lesions, they undergo a process of neuroangiogenesis (Asante and Taylor, 2011). This consists of the coordinated establishment of a blood supply through the generation of new blood vessels to support growth and survival (angiogenesis), together with the synchronized innervation by nerve fibers (neurogenesis) (Weinstein, 2005).

Neuroangiogenesis is regulated by estrogen and immune cells, including macrophages, which are an important source of vascular endothelial growth factor (VEGF) and nerve growth factor (NGF), both of which are increased in endometriosis (McAllister et al., 2009; Greaves et al., 2014). Macrophages exacerbate local inflammation, facilitate growth of ectopic endometrial lesions and are directly involved in angiogenesis, releasing chemokines and cytokines that further drive the growth of endometrial tissue (Hammond et al., 1993; Lin et al., 2006; Bulun, 2009). In a surgically transplanted endometriosis mouse model, macrophage secretion of the angiogenic factors VEGF, TNFα and macrophage inflammatory proteins (MIP-1α and MIP-2) into the peritoneal fluid in early stages of endometriosis progression correlated with a peak in active angiogenesis in lesions (Lin et al., 2006). Heightened angiogenesis, driving both the establishment and growth of endometrial lesions, is also evident in patients. Studies have identified increased angiogenic factors, including VEGF and TNFα, in the pelvic peritoneal fluid of patients with endometriosis (Shifren et al., 1996). These changes are further supported by a rich vascularization correlating with more active peritoneal endometrial lesions (Nisolle et al., 1993).

As angiogenic factors promote blood supply and support lesion growth and establishment, neurotrophic factors are essential for the development of both autonomic neurons and sensory afferent neurons, of which the latter can transmit nociceptive stimuli (Yan et al., 2017; Skaper, 2018). An increased density of small, unmyelinated nerve fibers (sensory afferents, sympathetic, and parasympathetic efferents) have been found in endometrial lesions (Tokushige et al., 2006b) and the endometrium of women with endometriosis (Tokushige et al., 2006a). The vast majority of these unmyelinated nerve fibers have been identified as C-fiber sensory afferents, unmyelinated nerves that typically function as nociceptors, implicating them strongly in the generation of CPP in endometriosis (Wang et al., 2009a). Directly highlighting their importance in CPP, early removal of lesions, before they established nerve fiber innervation, reversed hypersensitivity seen in an endometrial mouse model (McAllister et al., 2012). Moreover, endometriosis-associated menstrual pain was highest in women whose endometriotic lesions had increased nerve fiber innervation (Mechsner et al., 2009; McKinnon et al., 2012).

An increase in neuroangiogenic markers has been linked to a dense nerve supply in lesions and is closely related to CPP symptoms in women suffering from endometriosis (Kajitani et al., 2013; Morotti et al., 2014). Markers for both adrenergic and cholinergic neurons (neuropeptide Y), as well as unmyelinated C-fibers (substance P: SP, calcitonin gene-related peptide: CGRP) are significantly higher in the endometrium of patients with endometriosis (Kajitani et al., 2013). Increased levels of neurotrophins and their receptors, including NGF and its receptor tyrosine kinase A (TrkA), are also seen in endometrial biopsies of women with endometriosis (Tokushige et al., 2008). NGF promotes new nerve sprouting and acts directly on existing sensory nerve fibers to induce pain (Anaf et al., 2002; Chao, 2003). These effects are exacerbated by increased levels of circulating estrogen in women with endometriosis, as estrogen can enhance NGF activation of TrkA (Bjorling et al., 2002). This is important as its downstream target is the well-known nociceptive cation channel transient receptor potential subfamily V member 1 (TRPV1), which has been found to be upregulated in endometriomas and ectopic endometrial cells (Wang, 2008; Bohonyi et al., 2017). An increase in the density of nerve endings throughout lesions and an enhanced excitability of nerves provides the basis for increased nociception at lesion sites. The nerve injury-induced protein 1 (Ninj1), which promotes neurite outgrowth, has also been found to be expressed in ovarian and peritoneal endometriotic tissues (Miyashita et al., 2019).

Together with the establishment and growth of endometrial lesions, neuroangiogenesis aids irritation and invasion of existing nerves. Nerve density is increased in deep infiltrating endometriotic nodules when compared to healthy vaginal tissue, an observation also seen in peritoneal endometriotic lesions relative to the normal peritoneum (Tokushige et al., 2006b; Wang et al., 2009b; Anaf et al., 2011). The close proximity of endometrial adhesions and lesions to pelvic nerves can cause their encapsulation or compression, which contributes to CPP associated with endometriosis (Tulandi et al., 2001). Hyperalgesia due to dense nerve supply and encapsulation is supported by multiple studies, which show the highest pain score from endometriosis patients correlates with a higher density of nerves and nerve encapsulations (Anaf et al., 2000; Mechsner et al., 2009; McKinnon et al., 2012).

It has also been suggested that an imbalance between the activation of anti-nociceptive and pro-nociceptive ion channels on these lesion innervating neurons is a possible mechanism for pain pathophysiology (Arnold et al., 2012). Such imbalances also contribute to other visceral pain conditions (Sadeghi et al., 2018). An imbalance resulting in increased nociceptive input, with a decreased parasympathetic control at lesion sites could lead to a heightened pain response. Changes in this autonomic equilibrium occur in a number of chronic visceral pain disorders including fibromyalgia, gastroesophageal reflux disease and IBS (Aziz, 2013), with decreased parasympathetic and increased sympathetic activity (Heitkemper et al., 1998), favoring the transmission of painful stimuli. Sensory nerves had a significantly higher density in areas close to endometriotic lesions compared to sympathetic nerve fibers (Arnold et al., 2012). Interestingly, it has been shown that traditional hormone therapies that alleviate pain in endometriosis, including progestogens and oral contraceptives, significantly reduced nerve fiber density in ectopic endometrium (Tokushige et al., 2008). Although a novel finding, pain severity was not assessed, and further studies are needed to determine if the nerve fiber reduction in endometrial lesions in response to hormone therapy would show a clinical effect on lesion specific pain. Along these lines, estrogen receptor expression in endometrial samples has been shown to predict symptom severity and pain recurrence in endometriosis. In particular a higher expression of ER-α increased the prospect of moderate to severe dysmenorrhea and deep dyspareunia. Correspondingly, progestin therapy reduced ER-α expression, whereas androgen receptor, aromatase expression and progesterone receptor abundance were unaltered (Pluchino et al., 2020).

### Neurogenic Inflammation

Adding to the already pro-nociceptive environment induced by endometrial lesions in the peritoneal cavity, build-up of degraded tissue by-products, including ROS, PGE_2_ and acidification can directly act on or sensitize sensory nerve fibers through receptors on nociceptive afferent nerves located within lesions (Table 2 and Figure 1) (Reeh and Steen, 1996; Ma and Quirion, 2008; Holzer, 2011). Compounding this nociceptive barrage, sensitized sensory nerve fibers maintain inflammation by a positive feedback loop called ‘neurogenic inflammation.’ Excitation of these nerve fibers results in their terminals releasing further pro-inflammatory modulators. This includes neuropeptides such as SP and CGRP, both of which are found close to endometrial lesions in women with endometriosis associated pain (Tokushige et al., 2006a). Moreover, activation of sensory afferent nerves initiates the recruitment of mast cells and subsequent release of pro-inflammatory cytokines, including TNFα, NGF, PGE_2_ and a variety of interleukins, such as IL-1β, which contributes to a chronic state of neurogenic inflammation (van der Kleij et al., 2009; Chiu et al., 2012). This inflammation encourages further stimulation of locally circulating mast cells and macrophages that are found in elevated levels and in close proximity to nerve fibers in women with endometriosis (Bacci et al., 2009). A number of other chronic pain conditions, such as asthma, arthritis, interstitial cystitis, IBS, Inflammatory Bowel Disease (IBD; including Ulcerative Colitis and Crohn’s Disease), show a chronic neurogenic inflammatory state that induces chronic pain (Bordman and Jackson, 2006; van der Kleij et al., 2009; Brierley and Linden, 2014; Straub and Schradin, 2016).

### Peripheral Sensitization

Neuroplasticity of peripheral sensory nerves, such that structural, synaptic or intrinsic changes occur to shift neuronal function into a more sensitized state, is an established process in the development of chronic visceral pain (Brierley and Linden, 2014; Grundy et al., 2019). Under normal circumstances, peripheral sensitization causes the threshold for neuronal activation to drop, inducing pain from a stimulus that does not normally provoke pain (allodynia) or heightens existing pain (hyperalgesia). This mechanism provides protection from further damage following the development of inflammation from an existing injury (Iyengar et al., 2017). However, if inflammation persists, or a maladaptation to the original sensitizing stimuli occurs, nociceptors can become chronically hypersensitive even after inflammation resolves and histology appears normal. This peripheral hypersensitivity of nociceptive fibers at lesion sites may play a role in allodynia and hyperalgesia seen in endometriosis patients (Figure 1) (Morotti et al., 2017).

As described above, there is an abundance of inflammatory molecules, including glycodelin, ROS, TNFα, NGF and PGE_2_ in the peritoneal fluid of women with endometriosis, which may contribute to the induction of a more pro-nociceptive state (Table 2) (Chiu et al., 2012; St-Jacques and Ma, 2014). Increased levels of TNFα and glycodelin correlate with central hyperexcitability in response to repeated electrical stimulation and altered pain response to nociceptive withdrawal reflex, as well as higher levels of menstrual pain experienced by endometriosis patients (Scholl et al., 2009; Neziri et al., 2014). Patients with the highest pain scores also had the highest levels of TNFα, which was independent of lesion score (Overton et al., 1996). Further supporting a role in chronic pain conditions, TNFα activates nerve fibers innervating the colon, and has been shown to be elevated in samples from IBS patients, which correlates with patient clinical pain scores (Hughes et al., 2013).

The nociceptive ion channel TRPV1 has been implicated in other chronic pain conditions such as rheumatoid arthritis, osteo arthritis and IBS (Wang, 2008; Basbaum et al., 2009; Sadeghi et al., 2018), acting as a molecular sensor to potentiate and integrate responses to pain inducing stimuli, such as acidosis, oxidative stress or inflammatory mediators (Tominaga and Julius, 2000; Wang, 2008). Supporting an involvement of TRPV1 in endometriosis associated pain, elevated expression of TRPV1 has been found in dorsal root ganglion (DRG) of rats with endometriosis (Lian et al., 2017) as well as locally on infiltrating adhesions in endometriosis patients, the increase correlating with pain intensity (Rocha et al., 2011; Bohonyi et al., 2017). This is not surprising, as a number of the processes responsible for TRPV1 upregulation and sensitization are found in endometriosis patients, including enhanced ROS concentrations and increased levels of neurotrophins such as NGF. Together these mechanisms have the ability to activate TRPV1 as well as increase its expression, further driving sensitization of peripheral nociceptors (Wang, 2008; Ma et al., 2009; Kajihara et al., 2011). It has been proposed that chronic sensitization of peripheral sensory nerve fibers in turn induces sensitization of the CNS in endometriosis (Berkley et al., 2005; Arnold et al., 2012). Recent studies also show exposure to chronic stress early in life can increase the likelihood of CPP later in life, whilst bouts of acute stress can trigger or worsen symptoms (Pierce et al., 2014, 2015; Pierce and Christianson, 2015; Fuentes and Christianson, 2018).

### Central Sensitization

Chronic hyperexcitability of peripheral afferents has also been shown to induce long-term changes in CNS signaling, as well as increased sprouting of central terminals within the spinal cord dorsal horn in other painful visceral disorders, including IBS and interstitial cystitis (Harrington et al., 2012; Grundy et al., 2018, 2019). These conditions contribute to the sensitization of neurons in the CNS that produces long lasting hyperexcitability in the absence of noxious stimuli (Anaf et al., 2006; Schulke et al., 2009). The persistent nociceptive barrage from inflammatory endometriotic lesions is thought to lead to a long lasting central sensitization of sensory afferents (Figure 1) (Bajaj et al., 2003). This phenomenon has been seen in patients with chronic pain related to osteoarthritis, whiplash (Koelbaek Johansen et al., 1999) and fibromyalgia (Harris et al., 2009) and is often considered the cause for ‘unexplained’ chronic pain (Nijs et al., 2011).

Central sensitization can evoke long-term changes in pain processing, similar to the generation of ‘memory.’ Neonatal irritation of hollow organs or maternal separation are able to drive long-term visceral/somatic hypersensitivity and central sensitization (Pierce et al., 2014, 2015; Fuentes and Christianson, 2018). Central sensitization is also often initiated and maintained by peripheral sensitization, resulting in pain persisting long after the peripheral insult or pathology or has resolved (Table 2) (Woolf, 2011). This may be particularly relevant in endometriosis as many women experience persistent pain despite treatment or removal of endometrial lesions (Shakiba et al., 2008; Li et al., 2018). Furthermore, women with dysmenorrhea (menstrual pain) have increased CNS activation to noxious stimuli compared to women who don’t experience menstrual pain, a phenomenon seen at various stages of the menstrual cycle (Vincent et al., 2011). Accordingly, central sensitization of pain pathways could help to explain why chronic pain persists in some patients following lesion removal or why diagnosed lesion scores don’t correlate with pain intensity (Li et al., 2018). With this in mind, the location of the lesions, and their proximity to major peripheral nerves may provide an important but overlooked contribution to the development of chronic pain in patients diagnosed with endometriosis.

Neuroimaging has been used to identify chronic changes in the brains of women with CPP, both with and without endometriosis. A decreased volume of gray matter has been identified in areas of the brain related to pain processing, including the thalamus and insula, a brain region consistently activated during acute and chronic pain states (As-Sanie et al., 2016). These changes were seen in women with endometriosis that experience CPP but not in asymptomatic women, further supporting a role for CNS pain processing in CPP. Furthermore, proton magnetic resonance spectroscopy determined that women with endometriosis had increased levels of excitatory neurotransmitters in their anterior insula compared with healthy women (As-Sanie et al., 2016). Using an animal model of endometriosis, changes in gene expression in similar pain processing areas of the brain, including the amygdala, insula and hippocampus were also observed (Li et al., 2018).

Not only does central sensitization alter the pain response from the area of insult, it can also alter pain from seemingly unrelated areas. Animal models of endometriosis experience allodynia and hyperalgesia to different noxious stimuli including heat and vaginal distension (Nagabukuro and Berkley, 2007; McAllister et al., 2009; Li et al., 2018). Similarly, a cohort of endometriosis patients experienced enhanced muscular pain following saline injection into the hand when compared to healthy participants (Bajaj et al., 2003). In these scenarios, both hyperalgesia and allodynia are apparent once a stimulus is applied, allowing the changes in sensitization to become apparent. Although increased pain responses have been demonstrated in both animal and human studies, the CNS pathway changes, together with the molecular mechanisms underlying central sensitization in endometriosis, are more difficult to determine.

Although changes in central pain processing have been reported in endometriosis, whether they exacerbate CPP following changes initiated by endometriosis, or whether these women are already sensitized and have a heightened response to endometrial disease, is unclear. As the delay between onset of pain and diagnosis and treatment of endometriosis is typically between eight to 12 years, there is ample opportunity for these lesions to induce the chronic changes needed to induce central sensitization. Although it is currently unknown if earlier diagnosis and removal of lesions could reverse or avoid these changes in patients, the removal of lesions in the early stages of progression in animal models reversed the pain experienced (McAllister et al., 2009, 2012), whilst removing them at later stages had no effect (McAllister et al., 2012).

### Cross-Organ Sensitization

Women with endometriosis have a high comorbidity rate with other chronic pain syndromes associated with peripheral and central changes in pain processing, including, fibromyalgia, migraine headaches, IBS and painful bladder syndrome (Chung et al., 2002). The phenomenon of cross-organ sensitization, described as the spreading of noxious inputs from a diseased visceral organ to a normal organ in close proximity, has been studied in other pathological conditions where comorbidities are common, including IBS, IBD, interstitial cystitis and other CPP disorders (Wesselmann, 2001; Winnard et al., 2004, 2006; Pezzone et al., 2005; Grundy and Brierley, 2018).

Experimental evidence of functional cross-organ sensitization in the pelvis has been observed in many combinations, including bladder/colon (Qin et al., 2005; Grundy et al., 2018), vagina/colon, bladder/uterus (Ge et al., 2019) and uterus/colon (Lamb et al., 2006; Winnard et al., 2006). For example, uterine inflammation produced signs of inflammation in the colon and the bladder (Winnard et al., 2004), whilst bladder inflammation has been found to alter uterine contractility (Dmitrieva et al., 2001). These alterations have been displayed in preclinical endometriosis models where abnormal endometrial tissue growth induced vaginal hyperalgesia in a rat, which was exacerbated by estradiol (Berkley et al., 2007; Nagabukuro and Berkley, 2007). It has also been demonstrated that endometriosis increased pain behaviors of rats with kidney stones, another comorbidity of women with endometriosis (Giamberardino et al., 2002), and reduced the micturition threshold of rats, indicating increased urinary urgency and frequency, a clinical symptom of bladder disorders (Chung et al., 2002; Berkley, 2005).

Cross-organ sensitization is thought to occur when sensitized afferents from one organ induce sensitization of the afferents innervating another organ (Table 2 and Figure 2). Visceral afferents converge into similar areas of the spinal cord providing opportunity for the sensitization of neighboring cells due to spatial location (Grundy et al., 2018; Ge et al., 2019). The precise mechanisms responsible for cross-organ sensitization are unclear, however, overlap of peripheral afferent pathways within the DRG and spinal cord are crucial (Aredo et al., 2017; Grundy and Brierley, 2018; Grundy et al., 2019). Following neuroangiogenesis, which includes the presence of CGRP, TRPV1, and TRPA1 expressing nerve fibers (Fattori et al., 2020), the sensory nerves innervating endometriotic lesions may converge into the same spinal pathways from the afferents they originally sprouted from in the periphery. As such, they will share the same cell bodies in the DRG and the same central terminals within the spinal cord (Costa et al., 2004). As the location of ectopic lesions appears to be random, this could help to explain why the experience of pain is heterogenous within an endometriosis population.

With retrograde menstruation the primary theory for development of endometriosis, pelvic lesions are often found around the reproductive tract, with extra-pelvic lesions found in close proximity, located frequently upon the gastrointestinal tract and urinary system (Matalliotakis et al., 2017). Pain from these more distant lesions often mimics the pain from pelvic lesions and the extent of disease continues to be independent from symptom severity, regardless of lesion location. Conscious pain mapping of women with endometriosis confirmed half of participants localized their pain to lesion sites, but many women described generalized pain in the pelvis and bowel, indicating a widespread visceral hypersensitivity and pain independent of lesion location (Howard et al., 2000; Hsu et al., 2011). Palpation of various lesion types during laparoscopy of women with endometriosis also saw pain extend into normal looking tissue (Demco, 1998), whilst referred pain was reported in half of women under conscious sedation during laparoscopy for CPP (Demco, 2000). Lesion specific pain on visceral organs may be explained by neuroangiogenesis in lesions converging with existing neuronal pathways in the adhered organ, promoting cross-organ sensitization due to shared nerve pathways.

Cross-organ sensitization may also occur through shared innervation pathways of anatomically distinct organs. Recent studies have shown that a single peripheral neuronal cell body located within the DRG is able to generate multiple axons that innervate a number of abdominal organs simultaneously (Christianson et al., 2007; Chaban, 2008; Jobling et al., 2010). Often referred to as ‘dichotomizing afferents,’ these nerve fibers converge from multiple visceral organs to a single cell body within the DRG and therefore terminate onto the same second order spinal neurons. Whilst the evolution of these dichotomizing afferents is embedded within the physiological coordination of sexual, defecatory and urinary function, sensitization of these pathways in pathological conditions can allow cross-organ sensitization to occur. Retrograde tracing has revealed these neurons co-innervate a number of pelvic organs including the colon/bladder (Christianson et al., 2007; Grundy et al., 2018), colon/vagina (Ge et al., 2019), and colon/uterus (Chaban, 2008; Broad et al., 2009).

The chronic generalized hypersensitivity across visceral organs may in part be a result of cross-organ sensitization. The female reproductive tract is innervated by splanchnic, pelvic and pudendal nerve pathways, presenting an opportunity for cross talk through shared neuronal pathways of other organs in close proximity, such as the bladder and bowel. Whilst nerve development within lesions has been of interest, further understanding the path that new nerves take and the changes they facilitate is a necessary step in interpreting pain progression in endometriosis patients.

## Current Treatments

Eighty percent of endometriosis patients suffer from CPP, with subfertility affecting 60% of these women. Although both symptoms are prevalent, their treatment is often independent, with reproductive plans complicating optimal treatment regimens. With up to 87% of women presenting with CPP found to have endometriosis (Falcone and Flyckt, 2018), the treatment of CPP is an effective strategy to improve the quality of life in these patients, independent of subfertility. Overall, current therapeutic options provide pain relief for more than 6 months in only 40–70% of patients (Howard, 2000; Guo, 2009). As such, a greater understanding of the mechanisms underlying endometriosis induced pain is necessary to achieve greater clinical outcomes in the future.

As briefly mentioned above, traditional treatments focus on lesions as the cause of CPP associated with endometriosis (Table 2). Modulation of hormonal signaling via gonadotropin-releasing hormone (GnRH) agonists, aromatase inhibitors and oral contraceptive pills is known to suppress pain symptoms via estrogen inhibition and prevention of menstruation. However their incompatibility with fertility make them a short term treatment option for many women, with conclusion of treatment seeing the return of menstruation and symptoms (Kitawaki et al., 2002; Guo, 2009; Slopien and Meczekalski, 2016). By reducing the availability of estrogen, the estrogen dependent disease is significantly diminished, and cyclical bleeding is abolished. Although initially effective, pain relief was noted in only 40–70% of patients after 6 months (Howard, 2000). Together with the hypoestrogenic side effects including loss of bone mineral density and secondary osteoporosis, mood changes and breast atrophy (Kim et al., 2011), the use of GnRH is approved for only 6 months. Furthermore, contraceptive pills are not viable with pregnancy, making them a short-term solution for many women, especially those with reproductive plans. Aromatase inhibitors inhibit extra-ovarian estrogen production, making them particularly relevant for post-menopausal women or in combination with GnRH agonists, however, their reduction in pain is again offset by adverse side effects, including osteoporosis and bone fractures (Slopien and Meczekalski, 2016).

Non-hormonal pain reducing agents such as non-steroidal anti-inflammatory drugs (NSAIDs) and strong analgesics (opioids) reduce inflammation and mask pain (Brown et al., 2017). NSAIDs are used to target inflammation, although a review of their effectiveness in managing pain caused by endometriosis is inconclusive (Brown et al., 2017). Unfortunately, it has also been noted that NSAIDs are often ineffective and exhibit toxicity (Allen et al., 2005). Opioids therapy is also used to treat chronic pain associated with endometriosis and surgical intervention (Lamvu et al., 2019). Whilst opioids provide pain management, their effectiveness in the treatment of endometriosis is not reliably understood. Misuse and abuse is a growing problem amongst many chronic pain conditions, together with tolerance and addiction making opioids an undesirable treatment option (Kanouse and Compton, 2015). Unfortunately, whilst these medications represent a relatively non-invasive treatment plan, they either lack efficacy, or result in tolerance, dependence and addiction.

As the most invasive option, but often required due to the debilitating nature of chronic pain (Kanouse and Compton, 2015) and treatment of endometriosis associated subfertility, surgical treatment of endometriosis is based on the principal of removing all endometriotic lesions to alleviate symptoms. Whilst endometrial lesions within the peritoneum can be excised or ablated, they often grow back due to continuing retrograde menstruation and disease progression, and further surgery is needed in many cases (Cheong et al., 2008). Between 20–28% of patients are non-responsive to surgery (Sutton et al., 1994; Abbott et al., 2004), whilst reoccurrence is as high as 40–50% at 5 years post-surgery (Shakiba et al., 2008). The surgical removal of lesions can sometimes prove challenging depending on the stage and grade of endometriosis, which further complicates outcomes. Surgical removal of deep infiltrating endometrial lesions reduces endometrial CPP, but is associated with significant complication rates, requiring complex surgery to remove lesions, with resection of the bowel, damage to the bladder wall and stenting of the ureter often required (Wee-Stekly et al., 2015). The lowest rates of further surgery in pain management is following complete hysterectomy, with over 90% of patients remaining follow-up surgery free for up to 7 years, although the removal of the ovaries as well as the uterus is not as effective in younger women (Shakiba et al., 2008). In addition, associated health risks due to early menopause and incompatibility with pregnancy make hysterectomy a non-viable treatment for many women of reproductive age (Shakiba et al., 2008).

Even with a combination of therapies, complete eradication of endometrial associated pain is rarely possible. While the current therapies aim to suppress pain and delay reoccurrence, the disease often manifests itself once treatments have stopped or lesions have reformed. While the complete eradication of lesions is not likely, further understanding of chronic pain associated with endometriosis and identification of possible targets for pain management may help improve the quality of life for those suffering with endometriosis.

### Future Therapies? Insights From Animal Models of Endometriosis

Endometriosis is currently considered an inflammatory disease, with non-surgical treatment targeted at the prevention of menstruation and hormonal therapy to limit disease progression and inflammation. It is increasingly evident that this disease has a multifaceted nature with 40% of women experiencing non-cyclical pelvic pain, thus limiting endometriosis treatment to anti-inflammatory or hormonal control of menstruation seems to miss the mark. Currently the delay in diagnosis allows complex disease progression, increasing the opportunity for long term changes in pain processing to occur. Although progress has been made in the diagnosis and classification of endometriosis, assistance in early diagnosis is still of importance. Recent research priorities for endometriosis include an emphasis on biomarkers and imaging for non-invasive diagnostic techniques (Rogers et al., 2017). Despite multiple studies aimed at identifying a reliable biomarker as a clinical diagnostic tool, a systematic review of the last 25 years concluded that no single biomarker was confirmed to be clinically useful (May et al., 2010). Strikingly, it has also been noted that very little progress has been made in understanding chronic pain associated with endometriosis, and subsequent advancement in treatment is lacking (Rogers et al., 2017).

Modulating pain at an ion channel level and the targeting of specific pain receptors at lesion sites or organs involved in cross-organ sensitization may provide a novel and non-hormonal approach for endometriosis patients suffering from CPP (Castro et al., 2020). Indeed, limiting neuroangiogenesis to prevent lesion innervation and growth, together with direct modulation of key targets on peripheral nerves, is an important direction for future CPP management in endometriosis. Supported by evidence from a mouse model of endometriosis, lesion removal at early time points reduces CPP (McAllister et al., 2009, 2012; McKinnon et al., 2012). But in many cases this approach is not possible, whether it be delayed diagnosis, incomplete lesion removal, or deep infiltrating lesions, it is clear complete lesion removal at an early time point is a difficult venture.

Animal models of endometriosis have demonstrated novel mechanisms for reducing CPP. This includes the use of estrogen EP_2_ receptor antagonists to reduce primary hyperalgesia by 80% and secondary hyperalgesia by 40% in a mouse model of endometriosis (Greaves et al., 2014). Furthermore, bradykinin receptor 2 antagonists can block bradykinin induced endothelin-1 production, which is important as endothelin-1 causes neuronal sensitization (Yoshino et al., 2018). Other studies have demonstrated a key role for GPR30, a G protein-coupled receptor for estrogen, in endometriosis associated pain, whereby intra-lesion GPR30 agonists produce persistent mechanical hyperalgesia, whilst GPR30 antagonists inhibit mechanical hyperalgesia (Alvarez et al., 2014). Conversely, activation of cannabinoid receptor type 1 (CB_1_R), which are expressed by the sensory and sympathetic neurons innervating endometrial lesions, decreases endometriosis-associated hyperalgesia (Dmitrieva et al., 2010). Moreover, antagonists of CB_1_R increase endometriosis-associated hyperalgesia, suggesting an endogenous cannabinoid tone (Dmitrieva et al., 2010). More recent studies show that Δ9-tetrahydrocannabinol (THC) alleviates mechanical hypersensitivity and pain in a mouse model of surgically induced endometriosis. THC also restored cognitive function and inhibited the development of endometrial cysts (Escudero-Lara et al., 2020). These studies suggest CB_1_R plays a crucial role in the growth of lesions and endometriosis associated pain.

Recent studies have also suggested the potential of repurposing treatments for other conditions. This includes repurposing dichloroacetate, a pyruvate dehydrogenase kinase (PDK) inhibitor/PDH activator used for treating cancer. This is because dichloroacetate normalizes human peritoneal mesothelial cells metabolism, reduces lactate concentrations and endometrial stromal cell proliferation in a co-culture cell model. In a mouse model of endometriosis oral administration of dichloroacetate reduces lactate concentrations within peritoneal fluid and endometrial lesion size (Horne et al., 2019).

Animal models of other chronic pain conditions, such as IBS, have been used to successfully demonstrate the modulation of pain by targeting the periphery. For example chronic treatment with a guanylate cyclase-C (GC-C) agonist reversed the neuroplastic changes that cause both colonic hypersensitivity and cross-organ sensitization of the bladder in a mouse model of IBS (Grundy et al., 2018). More recently, the same agonist, whose direct target GC-C is expressed within the gastrointestinal tract, has been used to reduce vaginal hyperalgesia and allodynia in a rat model of endometriosis (Ge et al., 2019). This gives rise to the idea that targeting pain in one area may in fact reduce pain from other organs and that chronic changes due to an area of insult in one organ may in fact modulate pain experienced in another. Using animal models of endometriosis provides an important medium to explore the use of existing drugs for the treatment of pain associated with endometriosis and should continue to be utilized.

This review provides evidence for an overall heterogeneity in endometriosis, rather than a ‘one size fits all’ approach. This strongly suggests a personalized treatment approach based on etiology and symptomatology. Shifting the paradigm of lesion specific and cyclical inflammatory pain will continue to open up further areas to expand treatment opportunities. Utilizing animal models of endometriosis that closely recapitulate the human disease will provide an insightful opportunity to further study disease progression and chronic pain. With the development of animal models of endometriosis already providing quality insight into disease progression and current treatment mechanisms, the next step is to dig a little deeper, and elucidate disease specific changes and their targets at the lesion, DRG, spinal cord and brain levels.

## Author Contributions

All authors listed have made a substantial, direct and intellectual contribution to the work, and approved it for publication.

## Conflict of Interest

The authors declare that the research was conducted in the absence of any commercial or financial relationships that could be construed as a potential conflict of interest.
